# Infections Due to Carbapenem-Resistant Bacteria in Patients With Hematologic Malignancies

**DOI:** 10.3389/fmicb.2020.01422

**Published:** 2020-07-17

**Authors:** Rym Lalaoui, Emilie Javelle, Sofiane Bakour, Carles Ubeda, Jean-Marc Rolain

**Affiliations:** ^1^Aix Marseille Univ, IRD, APHM, MEPHI, Marseille, France; ^2^IHU-Méditerranée Infection, Marseille, France; ^3^Laveran Military Teaching Hospital, Marseille, France; ^4^Centro Superior de Investigación en Salud Pública, FISABIO, Valencia, Spain; ^5^Centers of Biomedical Research Network (CIBER), Epidemiology and Public Health, Madrid, Spain

**Keywords:** carbapenem resistance, bacteria, invasive infection, hematologic malignancies, antibiotic treatments

## Abstract

In developed countries, hematological malignancies (HM) account for 8 to 10% of cancers diagnosed annually and one-third of patients with HM (HMP) are expected to die from their disease. The former wide spectrum “magic bullet,” imipenem, has been ousted by the emergence of carbapenem resistant (CR) pathogens. In endemic areas, infections with CR-bacteria occur in vulnerable patients, notably in HMP, who suffer from high mortality related to infectious complications. In this work, we reviewed epidemiologic and clinical factors associated with CR-infections in adult HMP and data on CR-related mortality and antibiotic treatments in this population. We found that resistance profile of strains involved in HMP infections, mainly bacteremia, reflect local epidemiology. Significant risk factors for infections with CR-bacteria include sex male, age around 50 years old, acute leukemia, selvage chemotherapy, neutropenia, and digestive colonization by CR-bacteria. Mortality rate is high in HMP infected with CR-Enterobacteriaceae, more particularly in case of acute myeloid leukemia and unresolved neutropenia, due to inappropriate empiric management and delayed administration of targeted antibiotics, such as tigecycline, colistin, or new associations of active drugs. Thus, we developed an algorithm for clinicians, assessing the incremental risk for CR-bacterial infection occurrence and mortality in febrile HMP, to guide decisions related to empirical therapeutic strategies.

## Introduction

In economically developed regions of the world, 8 to 10% of the annual new cancer diagnoses account for hematological malignancies (HM) and one-third of the HM patients are expected to die of their disease (Chen and Chen, 2014; Errahhali et al., 2016; Batista et al., 2017; Medecine Matters, 2020)^1^. Lymphoid neoplasm represents about three-quarters of all HM, and within this group, mature B-cell malignancies dominate (source^2^) (Snowden et al., 2017).

Since 2001, the World Health Organization (WHO) has provided and updated consensus classification of HM based on cell lineage, genetic abnormalities, and clinical features, which defines over 60 disease subtypes in the latest 2016 edition (Arber et al., 2016). Diagnosis of HM is increasingly complex and relies on new sophisticated laboratory technologies that are not available worldwide. Therefore, disparities between geographical areas in terms of HM incidence and survival can result from: diversity among HM that complexifies standardized categorizations; heterogeneity of reporting systems, registries, and methodologies of reports; geographic and ethnic differences in endogenous and exogenous conditions at higher risk of HM occurrence, including genetic, toxic, and viral factors; variations in access to drugs and their metabolism (Rodriguez-Abreu et al., 2007; Chen and Chen, 2014; Errahhali et al., 2016; Li et al., 2016; Kirtane and Lee, 2017).

Besides the underlying patient’s conditions, the type and stage of the hematologic disease, acute intercurrent events such as infections can significantly modulate the prognosis. In a 14-year prospective longitudinal surveillance study conducted at a regional cancer center in United Kingdom, patients with HM had a 3-fold higher incidence of blood stream infections (BSIs) than patients with other cancers (Schelenz et al., 2013). Infection-related mortality in HM patients can be high due to weak immune systems and the accumulation of various factors such as neutropenia, excessive or repeated chemotherapy, long hospital stays, and hospital-acquired infections.

Since the spread of Gram-negative (GN) bacteria producing the extended spectrum ß-lactamase (ESBL) enzymes, which, in addition to penicillins, confer resistance to cephalosporins and monobactam (Trecarichi and Tumbarello, 2017), carbapenems have been used as last-line antibiotics to treat infection caused by this type of bacteria (Lalaoui et al., 2019). Unfortunately, shortly after the use of carbapenems, fermenters (Enterobacteriaceae), and non-fermenters (*Pseudomonas aeruginosa* and *Acinetobacter baumannii*), GN-bacteria rapidly developed resistance to this antibiotic family and spread throughout the world (Bialvaei et al., 2015). Several carbapenem resistance mechanisms have been developed by GN-bacteria, including the production of carbapenemase enzymes, which is nowadays identified worldwide (Nordmann and Poirel, 2014).

Regarding the major concern raised by the global expansion of carbapenem resistance associated with significant global mortality (Borer et al., 2009; Falagas et al., 2014), we focused on morbidity and mortality related to carbapenem resistance in adult patients with HM. We reviewed series and case reports of carbapenem-resistant (CR) GN-bacteria in this targeted population to describe the epidemiologic and clinical factors associated with the occurrence of CR infections. We collected data on antimicrobial susceptibility and the provided antibiotic treatments. We aimed at identifying key points that could help assess the risk of CR infection in HM patients and improve their survival.

## Methods

In this review, we reviewed series and selected case reports that reported colonization or infection of HM patients with CR bacteria. The electronic literature search was conducted in two electronic databases, Pubmed and Google Scholar. Papers published from January 2010 to January 2019 and written in English were included. A panel of keywords was used to target articles that address the topic of our review, including “bacteria,” “Gram negative,” “Enterobacteriaceae,” “*Klebsiella pneumoniae*,” “*Escherichia coli*,” “*Acinetobacter baumannii*,” “*Pseudomonas aeruginosa*,” “carbapenem resistance,” “carbapenemase,” “KPC,” “NDM,” “OXA,” “hematological malignancy,” “leukemia,” “colonization,” “infection,” “blood stream infection,” “bacteremia,” “neutropenia,” and “chemotherapy.” Only papers reporting cases of colonization or infection with CR bacteria in adult HM patients were selected. The following data were extracted from each study: study type, region, published year, study period, number of patients included, age, HM-type, CR-bacterial genera or species, sample, CR mechanism, antibiotic susceptibility profile of CR bacteria, comorbidity, significative risk factors associated with occurrence of CR bacteria, mortality associated with CR bacteria, significative risk factors of mortality associated with CR bacteria, and provided antibiotic treatments. A targeted search was performed to find the recommendations from medical societies for the management of neutropenic febrile patients and to document the use of new antibiotics to treat CR bacteria, selecting only the most recent references.

## Results and Discussion

**FIGURE 1:**
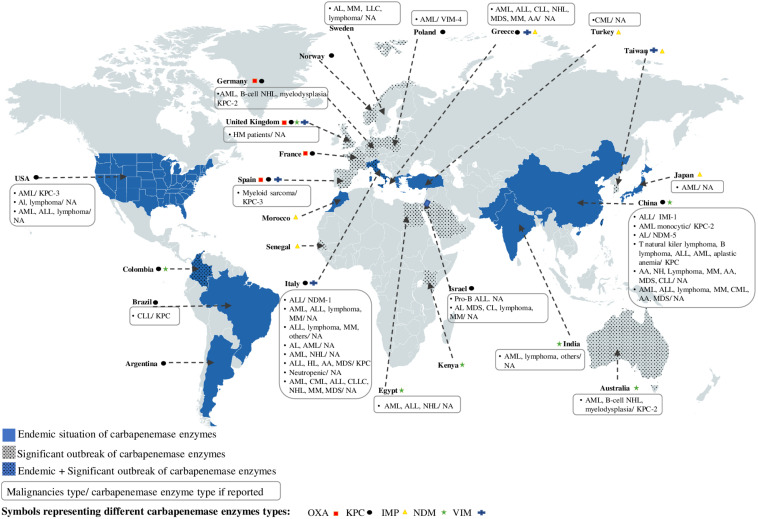
Worldwide distribution of carbapenemase enzymes depending on their regional endemicity or significant outbreak situation and geolocation of studies reporting the occurrence of CR bacteria isolated in HM patients. AA, aplastic anemia; AL, acute leukemia; ALL, acute lymphoid leukemia; AML, acute myeloid leukemia; CLL, chronic lymphocytic leukemia; CML, Chronic myeloid leukemia; HL, Hodgkin’s lymphoma; HM, hematologic malignancy; MDS, myelodysplatic syndrome; MM, myelome multiple; NHL, Non-Hodgkin’s lymphoma.

Based on our inclusion criteria, a total of 31 publications treating cases of colonization or infection by CR bacteria in adult HM patients were selected to conduct our review. Eighteen series (two prospective monocentric, three prospective multicentric, nine retrospective monocentric, and four retrospective multicentric) and 13 case reports published between 2012 and 2018 treated the topic that were conducted in 16 different countries, including: Australia (1/16), Brazil (1/16), China (6/16), Egypt (1/16), Germany (1/16), Greece (1/16), India (1/16), Israel (1/16), Italy (8/16), Japan (1/16), Poland (1/16), Spain (1/16), Sweden (1/16), Turkey (1/16), United Kingdom (1/16), and United States (3/16) (Figure 1; Tables 1−4). Depending on HM patient’s recruitment criteria used by the studies, 23/31 included cases of infections with CR bacteria, 2/31 included cases of colonization with CR bacteria, and 6/31 included both (Tables 1−4). Only 12 out of the 31 articles reported the CR mechanism (Figure 1; Tables 1−4). The production of carbapenemase enzymes was the only mechanism described with the predominance of KPC (*K. pneumoniae* carbapenemase) enzyme (8/12) followed by NDM [New Delhi metallo-ß-lactamases (MBL)] (2/12), VIM (Verona integron-encoded MBL) (1/12), and IMI (imipenem resistant variant 1) (1/12) enzymes (Figure 1; Tables 1−4). Thirteen publications reported some significative risk factors associated with occurrence of CR bacteria in HM patients (Table 1). The mortality rate associated with CR bacteria was indicated in 14 publications where 10 of them described also significative risk factors of mortality associated with CR bacteria (Tables 2, 3). Data on therapeutic management of CR bacteria in adult HM patients (neutropenic or not) were provided in only eight publications (Tables 2, 3).

**TABLE 1:** Characteristics of the studies reporting factors associated with CR bacteria in HM patients.

	**Study**	**Period**	**Place**	**Type**	**Recruitment**	**Global effective**	**CR Effective**	**Hematologic malignancies**	**Associated conditions**	**Significant risk factors for CR**
**Asia**	(Jaiswal et al., 2018) ***Mjhid***	10/2013–01/2016	New Delhi, India	prospective monocentric	consecutive newly diagnosed with HM hospitalized patients (no previous chemotherapy)	225	94/225 patients with CR-bacteria colonization (48/94 at admission) and 17/94 with CR-BSI	19 ALL, 37 AML, 22 lymphoma, and others	NA	AML, duration of hospitalization for CR-colonization, all CR-BSI occurred in CR-carriers
	(Wang et al., 2017) ***Eur J Clin Microbiol Infect Dis***	01/2014–06/2015	Jiangsu Province, China	retrospective multicentric (18 tertiary hospitals)	*A. baumannii* bacteremia in HM patients	3158 patients, 2133 GN isolates, 1358 BSI, 40 *A. baumannii-*BSI	13/40 *A. baumannii-*BSI	25 AL, 6 NHL, 4 MM, 2 AA, 2 MDS, 1 CLL	27/40 neutropenic, 16/40 central venous catheter	longer hospital stays, previous use of carbapenem, at least three types of antimicrobial therapy received before bacteremia
**Middle east**	(Andria et al., 2015) ***J Antimicrob Chemother***	2008–2014	Haifa, Israel	retrospective monocentric	GN BSI in HM patients	330 patients; 423 GN BSI	91/330 patients; 103/423 BSI	264/423 AL and MDS, 159/423 others (CL or lymphoma or MM)	364/423 nosocomial BSI, 388/423 central venous catheter, 53/103 carbapenem treatment 30 days before CR BSI	known CR carriage, salvage chemotherapy, urinary catheters, nasogastric tube, longer admission period, lower albumin level, hypotension
	(Kara et al., 2015) ***Infect Dis***	01/2005–12/2009	Ankara, Turkey	retrospective monocentric	BSI in HM patients	2098 HM patients, 281 with BSI representing 536 BSI episodes	11,5% of GN isolates	66 AML, 47 ALL, 6 MDS, 69 NHL, 37 MM, 14 HL, 13 CL, 7 AA, others	3703 neutropenic episodes	NA
**Northern europe**	(Ballo et al., 2019) ***PlosOne***	2007–2015	Frankfurt, Germany	retrospective monocentric	AML patients undergoing intensive induction chemotherapy	220 AML patients, 90/220 colonized at admission	12/90 patients colonized with CR-bacteria	AML	100% induction chemotherapy (cytarabine, daunorubicin) and levofloxacin 500 mg daily as prophylaxis	NA
	(Schlenz et al., 2013) ***JAC***	1997–2010	Norwich, United Kingdom	prospective monocentric	BSI in HM and solid cancer patients	HM patients: 473 BSI and 488 isolates; solid cancer patients: 441 BSI and 461 isolates	2, 5% among GN BSI in HM patients and less than 1% in solid cancer patients	NA	NA	NA
**Southern europe**	(Trecarichi et al., 2015) ***CMI***	2009–2012	Rome, Italy	prospective multicentric (9 hematology wards at tertiary care centers or at university hospitals)	consecutive HM patients bacterial BSI	575 healthcare and community-acquired BSI; 668 bacterial isolates; 344 GN	67 isolates	NA	529/575 neutropenic (92%)	NA
	(Pagano et al., 2014) ***Emerg Infect Dis***	01, 2009–12, 2012	Rome, Italy	retrospective monocentric	HM patients admitted to hospital with KPC-Kp BSI	147 GN BSI	26/147 GN BSI representing 26/38 *Kp*-BSI	14 AML, 4 NHL, 3 ALL, 2 HL, 1 AA, 1 MDS, 1 myelo-proliferative disease	13/26 initial or consolidation chemotherapy, 19/26 neutropenic	NA
	(Girmenia et al., 2015) ***Bone Marrow Transplantation***	01, 2010–07, 2013	Italy	retrospective multicentric (52 transplant centers)	HM patients undergoing HSCT	6058 auto-HSCT and 4389 allo-HSCT patients	CR-*Kp* colonization in 31 auto-HSCT and 51 allo-HSCT patients. CR-*Kp* infection in 25 auto-HSCT and in 87 allo-HSCT patients	40 AML, 18 ALL, 26 lymphoma, 8 MM, 20 others	84 neutropenia, 27 GVHD, CR-*Kp* previous colonization in 8 auto-HCST patients with CR-*Kp* infection and in 20 allo-HSCT patients	
	(Trecarichi et al., 2016) ***AJH***	01, 2010–06, 2014	Italy	prospective multicentric cohort study (13 hematological wardsof tertiary care centers or university hospitals)	hospitalized HM patients with *Kp* BSI	278 Kp-BSI	161/278	119 AML, 1 CML, 12 ALL, 1 CLL, 18 NHL, 3 MM, 2 MDS	129/161 chemotherapy, 71/161 corticosteroids	peripherally inserted central catheters (PICCs), AML, previous CR-*Kp* rectal swabs, antibiotic prophylaxis with fluoroquinolones
	(Micozzi et al., 2017) ***BMC ID***	02, 2012–05, 2013	Rome, Italy	retrospective monocentric	CR-*Kp* BSI in hospitalized HM patients	NA	22 patients with CR-*Kp* colonization and 14 with CR-*Kp* BSI	16 AL (12 AML) and others	5/22 recent carbapenem, 10/22 neutropenia, 20/22 chemotherapy	AML independent risk factor for bacteremia onset in carriers
	(Cattaneo et al., 2018) ***Annals of Hematology***	03, 2015–08, 2015	Italy	prospective multicentric (18 hematological institutions)	consecutive HM patients admitted to hematological wards	144 patients with MDR-bacteria rectal carriage incidence 6,5%	85/144 patients with CR-colonization, 12/85 with CR BSI	29/85 AML; 8/85 ALL; 32/85 lymphoma; 8/85 MM	12/85 salvage chemotherapy	for CR-BSI: urinary catheters, relapsed/refractory HM
**United States**	(Satlin et al., 2013a) ***Leukemia and Lymphoma***	07, 2007–12, 2010	New York, United States	retrospective monocentric	CR-BSI in HM patients	NA	18 patients	8 AL, 4 lymphoma or MM	13/18 neutropenia, 13/18 chemotherapy 16/18 prior systemic antibacterial therapy, 6/18 HSCT, 16/18 prior hospitalization	NA

*AA, aplastic anemia; AL, acute leukemia; ALL, acute lymphoid leukemia; allo-, allogenic; AML, acute myeloid leukemia; auto-, autologous; BSI, blood steam infection; CL, chronic lymphoma, CLL, chronic lymphocytic leukemia; CML, Chronic myeloid leukemia; CR, carbapenem-resistant; GN, Gram-negative;, GVHD, graft vs. host disease; HL, Hodgkin’ s lymphoma; HM, hematologic malignancy; HSCT, hematologic stem cell transplantation; Kp, Klebsiella pneumoniae; MDR, multidrug resistant; MDS, myelodysplatic syndrome; MM, myelome multiple; NHL, Non-Hodgkin’s lymphoma.*

**TABLE 2:** Characteristics of the studies reporting therapeutic management and mortality associated with CR bacteria in HM patients.

	**Study**	**Period**	**Place**	**Age (year)**	**CR Species**	**CR-Susceptibility (S) or resistance (R)**	**Therapeutic regimens**	**Mortality**	**CR-related mortality**	**Mortality risk Factors**
**Asia**	(Jaiswal et al., 2018) ***Mjhid***	10, 2013–01, 2016	New Delhi, India	46 and 49 [2–75] (medians [range])	*Kp* > *E. coli* colonization (mainly hospital acquisition), 12/17 BSI with *Kp* and 5/17 with *E. coli*	100% colistin S; 7/12 (58%) *Kp* and 4/5 (80%) *E coli* tigecycline S; 1/12 (8%) *Kp* and 1/5 (20%) *E. coli* aminoglycoside S	NA	9,5% global infection related mortality over 26 months	17/17 (100%) deaths in CR-BSI	AL, nosocomial acquisition
	(Wang et al., 2017) ***Eur J Clin Microbiol Infect Dis***	01, 2014–06, 2015	Jiangsu Province, China	55% <60	*A. baumannii*	100% colistin S, 91% tigecycline S and 76% amikacin S	Effective treatment in 20/40 (50%) *A. baumannii* BSI	32,5% at 30 days	8/13 (62%)	APACHE score, CR: independent risk factors for 30-day mortality in *A. baumannii* BSI
**Middle east**	(Andria et al., 2015) ***J Antimicrob Chemother***	2008–2014	Haifa, Israel	50 ± 15 (mean ± SD)	45% *Kp*, 25% *polymicrobial*, 15% *P. aeruginosa*, 13% *E. coli*, 13% S. *maltophila*, 9% *Acinetobacter* spp.	NA	35/103 (34%) appropriate empirical antibiotic treatment, 58/103 (56%) colistin	95/423 BSI (22%) at 14 days; 187/330 patients (57%) at 1 year	47/103 BSI (45,6%) at 14 days; 68/91 patients (74%) at 1 year	NA
**Europe**	(Ballo et al., 2019) ***PlosOne***	2007–2015	Frankfurt Germany	60,5 [18–85] (median, [range])	Enterobacteriaceae	NA	NA	8% at 2 months, 25% at 1 year and 41.5% at 2 years in colonized patients	33% at 2 months, 67% at 1 year and 75% at 2 years in CR-colonized patients	significantly reduced 60- and 90-day, as well as 1-and 2-year survival rates in CR-colonized patients (vs. non-colonized)
**Southern europe**	(Trecarichi et al., 2015) ***CMI***	2009–2012	Rome, Italy	52 ± 14 (mean, SD)	*15 Kp, 47 Pseudomonas* spp. *3 E. coli, 2 E. cloacae*	2/110 (2%) BGN tested colistin R (*Pseudomonas putida*), 9/160 (4%) tigecycline R (*Kp, E. cloacae, A. baumannii*)	NA	13% at 21 days	6/13 (46%) with CR-*Kp* vs. 3/20 (15%) with non CR-*Kp*; 14/19 (42%) with MDR *P. aeruginosa* vs. 2/16 (12%) with non-MDR *P. aeruginosa*	CR-GN BSI
	(Pagano et al., 2014) ***Emerg Infect Dis***	01, 2009–12, 2012	Rome, Italy	NA	*Kp*	81% colistin S, 69% tigecycline S, 65% gentamicin S	50% combined therapy with ≥2 active drugs	21% (32/147)	58% (15/26 KPC-*Kp*)	NA
	(Girmenia et al., 2015) ***Bone Marrow Transplantation***	01, 2010–07, 2013	Italy	54 in auto-HSCT group; 43 in allo-HSCT group (median)	*Kp*	NA	CR-*Kp*-targeted first line treatment in 6/25 (24%) auto-HSCT and 31/87 (36%) allo-HSCT patients, 11/112 tigecycline+ gentamicin, 3/112 colistin+ tigecycline+ gentamicin, 10/112 tigecycline+colistin	65/112	60/112	not CR-*Kp*-targeted first-line antibiotic therapy
	(Trecarichi et al., 2016) ***AJH***	01, 2010–06, 2014	Italy	57% ≥55	*Kp*	NA	NA	101/278 (36%) at 21-days	84/161 (52%) at 21 days	CR BSI, septic shock, inadequate initial antimicrobial therapy
	(Micozzi et al., 2017) ***BMC ID***	02, 2012–05, 2013	Rome, Italy	54 [28–76] (mean, [range])	*Kp*	12/22 colistin S, 6/22 tigecycline S and 0 gentamicin S	9/14 (64%) at least one drug active, 6 of which empirically	12/22	10/22 deaths due to CR-BSI	AML
	(Cattaneo et al., 2018) ***Annals Hematology***	03, 2015–08, 2015	Italy	60 [0–89], (median [range])	12 CR-BSI*:* 8 *Kp* 1 *Enterobacter sp.;* 2 *Acinetobacter Sp.;* 1 *P. aeruginosa*	NA	NA	10/144 (7%) at 30-days	5/10 (50%) deaths due to CR BSI	NA
**United States**	(Satlin et al., 2012) ***Leukemia and Lymphoma***	07, 2007–12, 2010	New-York, United States	56 [24–77] (median [range])	14 *Kp*, 3 *E. cloacae*, 1 polymicrobial	80% colistin S, 65% tigecycline S, 30% gentamicin S	2/18 adequate empirical therapy 10/15 one active drug 5/15 two active drugs	NA	10/18 (56%): 7 at 7 days, 9 at 14 days; 3 deaths before susceptibility data	NA

*AL, acute leukemia; allo-, allogenic; AML, acute myeloid leukemia; auto-, autologous; BSI, blood steam infection; CR, carbapenem-resistant; GN, gram- negative; HSCT, hematologic stem cell transplantation; Kp, Klebsiella pneumoniae; MSR, multidrug resistant; R, resistant; S, sensitive.*

**TABLE 3:** Characteristics of the studies reporting CR bacteria in HM neutropenic patients.

**Study**	**Period**	**Place**	**Type**	**Recruitment**	**Global effective**	**CR Effective**	**HM**	**CR Species**	**CR- Suscept ibility (S)**	**Thera -peutic regimens**	**Mortality**	**CR- Mortality**	**Mortality risk factors**
(Wang et al., 2015) ***Medicine***	2008–2014	China	retrospective monocentric	BSI in febrile neutropenic HM patients undergoing HSCT	273 patients, 348 episodes of neutropenic fever, 85 neutropenic patients with BSI, 89 episodes of BSI, 108 isolates	12/108 (11%) isolates, 12/73 (16%) GN isolates	22 AML, 16 ALL, 13 lymphoma, 4 MM, 2 CML, 2 AA, 2 MDS, 4 others	4 *Kp* (16.7%), 3 *S. maltophilia*, 1 *P. aeruginosa, 1 C. freundii*, 1 *P. stutzeri*, 1 *A. baumannii* and 1 *C. indologenes*	NA	NA	11/85 (13%) patients with BSI cause of death	6/11 (55%) BSI-related deaths due to CR	BSI with CR-bacteria and prolonged neutropenia (≥15 days, RR 16.7)
(El-Mahallawy et al., 2014) ***J Egypt Natl Canc Inst***	01, 2009–12, 2009	Cairo, Egypt	retrospective monocentric	febrile neutropenia among HSCT recipients	90 febrile neutropenia episodes in 50 patients, 39 BSI, 26 GN isolates	20% of GN bacteria	24 AML, 12 ALL, 7 NHL, and 7 other HM	NA	NA	NA	5/50 (10%) with 60% infection attributed	NA	NA
(Kjellander et al., 2012) ***Eur J Haematol***	2002–2008	Stockholm Sweden	retrospective monocentric	BSI in HM patients during chemotherapy-induced neutropenia	677 BSI episodes in 463 patients	6 (1%) isolates	1/3 lymphoma, 1/3 AL, 1/3 MM or CLL	1 *Enterobacter* spp. and 5 *P. aeruginosa*	100% gentamicin S	NA	5.2% at 7 days 13.6% at 30 days	NA	NA
(Tofas et al., 2016) ***IJAA***	2010- 2014	Athens, Greece	retrospective multicentric (4)	neutropenic HM patients with CR-*Kp* BSI	NA	50 patients	34 AML, 6 ALL, 1 CLL, 5 NHL, 1 MDS, 1 MM and 2 AA	CR-Kp bacteremia	90% gentamicin S, 86% tigecycline S, 74% colistin S	33/50 (66%) empirical treatment with ≥one active drug, 40/50 (80%) definitive treatment with ≥one active drug, 30/50 combination therapy	50% at 14 days	50% at 14 days	unresolved neutropenia, septic shock, inadequate treatment, treatment with one active drug vs. combination
(Satlin et al., 2016) ***J infect***	2008–2012	New York, United States	retrospective multicentric case control	neutropenic HM patients	1992 BSI	43 CR BSI (2%)	24 AML, 8 ALL, 8 lymphoma, 3 others 15 allo-HSCT, 3 auto-HSCT	30 *Kp*, 8 *E. cloacae*, 2 *E coli, 1 E. aerogenes, 1 E. gergoviae, 1 K. oxytoca* and 1 *S. marcescens*, 11 polymicrobial	tigecycline 86% S, polymyxin B 82% S, amikacin and gentamicin 48% S	6/43 (14%) active empirical treatment within 12h (vs. 56 to 88% in controls), median 52h [IQR 33-69] from BSI onset until active therapy	NA	22/43 (51%) at 30 days vs. 15% in controls (*p* < 0.001)	CR-inactive empirical therapy

*AA, aplastic anemia; AL, acute leukemia; ALL, acute lymphoid leukemia; allo-, allogenic; AML, acute myeloid leukemia; auto-, autologous; BSI, blood steam infection; CLL, chronic lymphocytic leukemia; CML, Chronic myeloid leukemia; CR, carbapenem-resistant; GN, Gram-negative;, HM, hematologic malignancy; HSCT, hematologic stem cell transplantation; Kp, Klebsiella pneumoniae; MDS, myelodysplatic syndrome; MM, myelome multiple; NHL, Non-Hodgkin’s lymphoma, S, sensitive.*

**TABLE 4:** Characteristics of case reports studies reporting CR bacteria in HM patients.

	**Study**	**Place**	**Year**	**Age (Y)**	**Hematologic malignancies**	**Other conditions**	**Therapeutic antiobiotic administrated**	**CR Species**	**Sites of isolation**	**Resistance mecanisms**	**Antimicrobial susceptibility**	**Death**
**Asia**	(Asai et al., 2018) ***Infectious Diseases in Clinical Practice***	Japan	NA	55	AML	neutropenia 5 days after HSCT	tigecycline	*Kp*	stool, blood	*bla*_*DHA–1*_ and Plasmid FIA(HI1)	tigecycline, colistin	Yes
	(Huang et al., 2015) ***Ann Clin Microbiol Antimicrob***	China	2014	30	ALL	previous exposure to colistin	NA	*E. cloacae*	blood	IMI-1 variant	ceftazidime, amikacin, ciprofloxacin, tigecycline, aztreonam, ceftriaxone, cefepime, piperacillin–tazobactam, gentamicin, tobramycin, levofloxacin, trimethoprim, minocycline	NA
	(Zhang et al., 2018) ***Emerging Microbes and Infections***	China	2014–2017	29	AML monocytic	NA	meropenem, isepamicin, vancomycin, teicoplanin, piperacillin-sulbactam, tigecycline, fosfomycin, amikacin, colistin, linezolid, polymyxin B	*Kp* (17 isolates)	fecal, blood, sputum	KPC-2	tigecycline in strains 1 to 8, colistin in strain 1 to 9	No
	(Zhang et al., 2016) ***IJID***	China	2014	56; 22; 63	AL	imipenem and vancomycin prophylaxis	NA	*E. coli*	blood	NDM-5	NA	NA
	(Xing et al., 2015) ***Internal Medicine***	China	2014–2016	40; 54; 59; 58; 44	T natural killer (NK) lymphoma, B lymphoma, ALL, AML, AA	1/5 agranulocytosis	Moxifloxacin–sulbactam/cefoperazone–tigecycline–fosfomycin–imipenem/cilastatin; meropenem–tigecycline; tigecycline–cefoperazone/sulbactam; moxifloxacin–amikacin; imipenem/cilastatin–vancomycin–tigecycline–amikacin–fosfomycin	*Kp*	4 blood, 1 sputum	KPC -2	100% tigecycline, 1/5 levofloxacin, 3/5 amiklin, 2/5 gentamicin, 2/5 vibramycin	4/5
**Middel east**	(Kantarcioglu et al., 2016) ***Molecular and Clinical Oncology***	Turkey	2015	50	CML	prior hospitalization in Libya	tigecycline, meropenem, imipenem, gentamicin, sulbactam,	*Kp*	blood and valve	NA	tigecycline	No
	(Muchtar et al., 2012) ***IMAJ***	Israel	2012	64	pro-B ALL	neutropenia	piperacillin/tazobactam, vancomycin, meropenem, tigecycline, rifampicin and amikacin	*Kp*	blood, skin abscess	NA	colistin, amikacin	Yes
**Northern europe**	(Majewski et al., 2017) ***IJID***	Poland	2013	60	AML	neutropenia, trimethoprim and aciclovir prophylaxis	cefepime, amikacin, linezolid, teicoplanin, colistin, meropenem	*C. freundii*	urine	VIM-4	colistin	Yes
	(Leitner et al., 2014) ***AAC***	Austria	2011-2013	[39-89]	7 AML, 2 B-cell NHL, 1 myelodysplasia	NA	NA	*K. oxytoca*	blood, skin, stool, urine, abscess, bronchoalveolar lavage, throat, swab	KPC-2	amikacin, colistin fosfomycin	4/11
**Southern europe**	(Carattoli et al., 2013) ***Euro Surv***	Italy	2013	40	ALL	prior hospitalization in Belgrade	piperacillin/tazobactam, amikacin, vancomycin, meropenem, colistin, tigecycline, rifampicin, daptomycin	*P. aeruginosa*	perianal abscess, blood	NDM-1 ST235 strain	colistin	Yes
	(Piedra-Carrasco et al., 2017) ***Microbial Drug Resistance***	Spain	2015	36	Myeloid sarcoma	ciprofloxacin prophylaxis	piperacillin/tazobactam, amikacin, double- carbapenem regimen ertapenem + imipenem	*Kp*	urine	KPC-3	gentamicin, colistin, tigecycline	No
**America**	(Satlin et al., 2013b) ***JCM***	United States	NA	70	AML	neutropenia	piperacillin/tazobactam, vancomycin, meropenem, trimethoprim- sulfamethoxazole, linezolid, amikacin	*E. gergoviae*	blood	KPC-3	levofloxacin, gentamicin, amikacin, tigecycline	Yes
	(Chang et al., 2013) ***Rev Soc Bras Med Trop***	Brazil	2010	94	CLL	NA	ciprofloxacin, piperacillin/tazobactam, vancomycin	*Kp*	Urine	KPC-2	cefoxitin, cefepime, colistin, tigecycline	Yes

*AA, aplastic anemia; AL, acute leukemia; ALL, acute lymphoid leukemia; AML, acute myeloid leukemia; CML, Chronic myeloid leukemia; Kp, Klebsiella pneumoniae; NHL, Non-Hodgkin’s lymphoma.*

### Epidemiology of Gram-Negative/Gram-Positive Infections Occurrence in HM Patients

There are epidemiologic specificities per regions of the world in the predominance of GN or Gram-positive (GP) causal pathogens responsible for (BSIs) in HM patients. Indeed, 62% of BSIs in HM patients in the United Kingdom were documented with GP strains (Schelenz et al., 2013), and such GP predominance was reported from Northern Europe and the United States (Kjellander et al., 2012; Schelenz et al., 2013; Satlin et al., 2016). Conversely, GN isolates were responsible for more than 50% of BSIs in HM patients in Italy (Trecarichi et al., 2015), Turkey (Kara et al., 2015), Egypt (El-Mahallawy et al., 2014), and China (Wang et al., 2015). Therefore, the proportion of all CR pathogens is not comparable to the part of GN bacteria in areas with different epidemiology. These data highlight the need for clearer and more standardized measures, which is why we have been as exhaustive as possible in the reports of the studies’ results in this review.

### Carbapenem Resistance in Hematological Patients vs. the World *Geographical comparison*

The emergence of resistance to carbapenems (imipenem, meropenem, ertapenem) is one of the most important problems with antibiotic resistance because there are few possible therapeutic alternatives. The resistance of GN bacteria to carbapenems may be due to several different mechanisms that confer a variable rate of resistance to this family of antibiotics, depending on how these mechanisms are expressed, that is, alone or in combination. This resistance can be due to structural mutations that affect the expression of some specific components of the membrane, such as efflux pump(s) and/or porins; a combination of these structural damages to the production of ESBLs or cephalosporinases; modification at the level of penicillin binding protein inducing a decrease in their sensitivity to the carbapenem molecules; and finally it can be due to the production of carbapenemase enzymes (Vila et al., 2007; Hui et al., 2013; Logan and Weinstein, 2017).

The production of carbapenemase enzymes is the most widespread mechanism of carbapenems resistance in the world, and their spread was mainly related to the GN bacteria, especially to Enterobacteriaceae. Based on the Ambler classification system, the carbapenemase belong to three different classes of β-lactamases: class A, class B, and class D β-lactamases.

Class A carbapenemase was either chromosomal encoded such as NmcA (non-metallo-carbapenemase-A), Sme (*Serratia marcescens* enzyme), IMI-1, and SFC (*Serratia fonticola* carbapenemase), or plasmid encoded such as GES (Guiana extended spectrum) and; KPC enzyme, which constitute the most transmissible and circulating class A carabapenemase enzyme in Enterobacteriaceae (Pfeifer et al., 2010; Logan and Weinstein, 2017). Unlike classes A and D β-lactamase, which require serine at their active site, class B β-lactamase or MBL, require zinc to hydrolyze β-lactam (Logan and Weinstein, 2017). VIM, imipenem (IMP), and NDM enzymes are the MBL enzymes most often detected in GN bacteria (Pfeifer et al., 2010; Logan and Weinstein, 2017). Finally, the class D carbapenemases, also known as oxacillinases, have been subdivided into different subgroups, some of which were of great clinical importance due to their wide acquisition by pathogenic bacteria. These important subgroups are mainly represented by oxacillinase (OXA)-23, OXA-24/40, OXA-48, OXA-51, OXA-58, and OXA-143 subgroups (Pfeifer et al., 2010; Antunes et al., 2014; Nordmann and Poirel, 2014). This family has minimal activity on carbapenems compared to that of the KPC or NDM enzymes, except in the case where these enzymes are associated with other factors such as membrane impermeability (Nordmann and Poirel, 2014).

We have represented in Figure 1, the geographical distribution of the clinically important carbapenemase enzymes found in Enterobacteriaceae family, including OXA, KPC, IMP, NDM, and VIM enzymes. As it can be seen, in addition to sporadic cases, carbapenemase enzymes were found to be endemic or significantly spread in different continents of the world, such as North America (United States), South America (Colombia, Brazil, Argentina), Africa (Morocco, Egypt, Kenya, Senegal), Europe (Spain, France, United Kingdom, Germany, Poland, Norway, Sweden, Italy, and Spain), Asia (Turkey, Israel, India, China, Taiwan, Japan), and Australia (Figure 1).

In order to identify the occurrence of CR in HM patients and to understand its epidemiology, we reviewed series and selected case reports that report colonization or infection of HM patients with CR bacteria. These studies were undertaken in different areas of the world, including New York, New Delhi, China, Stockholm, Frankfurt, Roma, Brescia, Athens, Ankara, Cairo, and Haifa in the series studies (Tables 1−3; Cattaneo et al., 2012, 2018; Kjellander et al., 2012; Schelenz et al., 2013; Satlin et al., 2013a, 2016; El-Mahallawy et al., 2014; Trecarichi et al., 2015, 2016; Wang et al., 2015, 2017; Micozzi et al., 2017; Ballo et al., 2019), and in United States, Brazil, Spain, Italy, Poland, Turkey, Israel, Japan, China, and Austria in the case reports (Table 4; Muchtar et al., 2012; Carattoli et al., 2013; Chang et al., 2013; Satlin et al., 2013b; Girmenia et al., 2015; Huang et al., 2015; Kara et al., 2015; Leitner et al., 2015; Xing et al., 2015; Kantarcioglu et al., 2016; Tofas et al., 2016; Zhang et al., 2016, 2018; Majewski et al., 2017; Piedra-Carrasco et al., 2017; Asai et al., 2018). When we geolocate the regions that have reported the presence of CR bacteria in HM patients in Figure 1, it can be observed that all these studies have been reported in regions that are either endemic or have a significant increase in carbapenemase enzymes (a coincidence with regions with high levels of CR bacteria) (Figure 1).

For example, China and Italy are the two countries with the highest number of studies reporting cases of colonization or infection with CR bacteria in adult HM patients. The CR mechanism treated in these articles report the presence of the KPC (China and Italy), NDM (China and Italy), or IMI (China) enzyme type (Figure 1).

From the viewpoint of the epidemiology of healthcare-associated infections, China and Italy are both endemic to at least two carbapenemase enzymes, namely KPC and VIM (Italy) or KPC and NDM (China). This supports our hypothesis that there is a relationship between the epidemiology of healthcare-associated infections and that one is found in MH patients.

It should be noted that in some areas of the world, where carbapenemase enzymes are either endemic (Argentina) or present a significant outbreak (Australia, Taiwan, Norway, France, Morocco, Senegal, Kenya, and Arabia Saudi) or both, as in Colombia, no data on the presence of CR bacteria in HM patients have been reported (Figure 1). Therefore, the implementation of diagnostic and therapeutic tools, together with a monitoring system for CR in hematological units in these regions, is necessary.

The CR mechanisms of bacteria detected in HM patients are very rarely studied. Apart from the case reports that indicate this information, the series are more focused on the clinical aspect of the resistance in these patients (comorbidity, risk factors, mortality, and therapeutic strategies). The analysis of data extracted from case reports showed that the KPC enzyme was the predominant carbapenemase enzyme detected in CR bacteria isolated from HM patients (Table 4; Chang et al., 2013; Satlin et al., 2013b; Leitner et al., 2015; Xing et al., 2015; Piedra-Carrasco et al., 2017; Zhang et al., 2018). When we compare this data with those related to the global distribution of carbapenemase worldwide, it is clear that the occurrence of the KPC enzyme in HM patients is related to its presence in the area (endemic or significant outbreak state) where the studies were conducted (China, Italy, Brazil, United States, Spain, Germany). Other carbapenemase enzymes have also been identified in CR bacteria isolated from HM patients, such as IMI (Huang et al., 2015), VIM (Majewski et al., 2017), or NDM (Carattoli et al., 2013; Zhang et al., 2018).

Overall, it appears that the resistance profile of strains involved in BSIs in patients with HM generally reflects the epidemiology of the country itself, with the exception of local outbreaks (Trubiano et al., 2015).

#### Etiology of CR-Bacteria in HM Patients

In clinical settings, *K. pneumoniae* is considered an important opportunistic GN-pathogen, responsible for several nosocomial infections such as pneumonia, septicemia, and infections in newborns and intensive care patients (Pitout et al., 2015). In HM patients, this species is the most widespread etiological bacterium in the world, detected in infections occurring in this group (Tables 2–4). CR *K. pneumoniae* was detected mainly as rectal colonization (Cattaneo et al., 2018; Jaiswal et al., 2018), CR-bacteria BSIs (Satlin et al., 2016), and nosocomial CR infection cases (Hussein et al., 2013; Cristina et al., 2017; Jaiswal et al., 2018).

In Europe, several studies have reported the occurrence of CR bacteria, mainly represented by *K. pneumoniae* followed by *Klebsiella oxytoca*, *Citrobacter freundii*, and *P. aeruginosa* (Tables 1−4). Most of the investigations conducted on CR bacteria in HM patients were undertaken in Italy. Indeed, in this region, when we reviewed the long-term prospective or retrospective studies (2009–2019, 2010–2012, 2010–2014) (Tumbarello et al., 2014; Trecarichi et al., 2015, 2016), we found that CR *K. pneumoniae* was the bacterial species most frequently responsible for infections (mainly BSIs) in HM patients identified at different rates [12.4% (Tumbarello et al., 2014), 35% (Trecarichi et al., 2015), and 57.9% (Trecarichi et al., 2016)]. In addition to CR *K. pneumoniae*, 70% of multidrug-resistant (MDR) *P. aeruginosa*, including those resistant to carbapenem involved in BSIs of HM patients, were reported in Italy (Cattaneo et al., 2012; Trecarichi et al., 2015).

In America, a few studies have reported the emergence of CR bacteria in HM patients. Most of them were undertaken in North America and more precisely in New York (Figure 1; Table 4). The first study reported the occurrence of BSIs in neutropenic patients with acute myeloid leukemia (AML) due to the *Enterobacter gergoviae* strain producing the KPC-3 carbapenemase enzyme. This strain was nevertheless sensitive to levofloxacin, gentamicin, amikacin, and tigecycline (Table 4; Satlin et al., 2013b). *E. gergoviae* species is among the least common CR pathogens responsible for BSIs reported in neutropenic patients. The second study was a retrospective multicentric case control conducted over a 4-year period (2008–2012) on 1992 BSIs cases that occurred in neutropenic HM patients, where 43 cases (2.2%) of BSIs were reported to be due to CR bacteria, with the *K. pneumoniae* species predominating (68% of CR BSIs), followed by *Enterobacter cloacae* (18% of CR BSIs), *E. coli* (5% of CR BSIs), and others (Satlin et al., 2013a). The last study was a retrospective monocentric analysis that included 18 febrile neutropenic HM patients with CR BSIs, and as expected, the most frequently found etiological bacterium responsible for BSIs in these patients was *K. pneumoniae* (14 out of 18) followed by *E. cloacae* (3 out of 18) (Satlin et al., 2013a; Table 4). Only one study was conducted in Brazil (South America), in which the authors reported a KPC-producing *K. pneumoniae* collected during a urinary tract infection in a patient with chronic lymphocytic leukemia (Chang et al., 2013).

Unlike other continents, the etiology of CR related to HM patients in Asia is more diverse. When we examined the series and cases reports, different CR-bacterial species were detected in HM patients, including *K. pneumoniae* (Wang et al., 2015; Xing et al., 2015; Asai et al., 2018; Zhang et al., 2018), *E. cloacae* (Huang et al., 2015), *E. coli* (Zhang et al., 2016), and *A. baumannii* (Wang et al., 2015). These bacteria have been isolated either in a context of colonization (stool, sputum) or infection (mainly BSIs).

Neutropenia is the predominant condition in HM patients reported in the reviewed studies. CR BSIs in these patients have also been documented with less-frequent pathogens, such as *E. gergoviae*, *K. oxytoca*, *S. marcescens* (Satlin et al., 2016), *Stenotrophomonas maltophilia*, *C. freundii*, *Pseudomonas stutzeri*, and *Chryseobacterium indologenes* (Wang et al., 2015).

### Risk Factors for CR Bacterial Infection in HM Adult Patients

#### Literature Review

In several areas, HM was reported as a significant risk factor for CR-Enterobacteriaceae positive culture, representing a comorbid condition in 8 to 27% of the hospitalized documented cases (Tumbarello et al., 2014; Marimuthu et al., 2017). In the 14-year prospective longitudinal surveillance study conducted in the United Kingdom, 2.5% of the GN pathogens isolated from bacteremia in HM patients were resistant to meropenem, compared to less than 1% in patients with solid cancer (Schelenz et al., 2013). In a prospective Italian cohort study conducted in nine hematology wards in 2009 to 2012, 21% of GN bacteria isolated from patients with BSIs (among 668 bacterial isolates) were resistant to meropenem. In a retrospective monocentric Turkish study on BSIs in HM patients over a 5-year period, 12% of GN-bacteria were resistant to meropenem (Kara et al., 2015).

Among all HM, the most at-risk population was on average 50 years old, mainly men, with acute leukemia (AL) or relapsed/refractory hematological disease (Tables 1−2). One study reported an age beyond 60 years as a major risk factor and lymphoma as protector (Cattaneo et al., 2018). Factors significantly associated with CR infections were longer hospital stays before bacteremia, CR-bacteria carriage, previous CR isolate, urinary catheters, and AML (Table 1; Satlin et al., 2016; Marimuthu et al., 2017; Micozzi et al., 2017; Wang et al., 2017).

In hematological cohorts without specific recruitment, white blood count, central venous catheter, and previous antibacterial therapy, including recent carbapenem treatment or antibio-prophylaxis (mainly fluoroquinolones), were inconstant risk factors for the occurrence of invasive infection with CR bacteria (Andria et al., 2015; Micozzi et al., 2017; Cattaneo et al., 2018), although these were frequent associated conditions (Table 1; Trecarichi et al., 2015; Satlin et al., 2016; Tofas et al., 2016; Wang et al., 2017). This is in line with emerging data that tend to put antibiotic pressure in the background to explain the emergence of resistance (D’Costa et al., 2011). However, some regions of the world with the highest prevalence of CR bacteria (e.g., Asia, Middle East, North Africa, Central and South America) clearly do not overlap with regions of the world with the greatest access to and use of carbapenems in community-acquired infections (Righi et al., 2016; Versporten et al., 2018). In a case-control study investigating risk factors for infection or colonization with CR *K. pneumoniae* conducted in Philadelphia, very few patients had a history of carbapenem use (1%) (Gasink et al., 2009). Antibiotic resistance is probably not only selected by direct pressure during use but also results from complex interactions in natural populations and ecosystems (Hiltunen et al., 2016). Moreover, a major bias of these studies is the restriction to healthcare-associated infections, and systematic exclusion of community acquired invasive infections, although community carriage is increasingly documented. This nosocomial recruitment obviously overestimates the role of prior exposition to antibiotics in the CR-bacteria sepsis occurrence.

A salvage chemotherapy was also associated with the occurrence of CR-bacterial infection (Andria et al., 2015). We hypothesize that the antibacterial properties of sequential chemotherapies could have an impact on the microbiomes of treated patients and alter the natural barriers that prevent endo-invasive infections with bacterial colonizers. Indeed, Galloway-Peña et al. (2016) highlighted in a prospective cohort of adults undergoing induction chemotherapy for AML, that a decrease in the Shannon diversity index of both oral and stool samples was a significant risk factor for subsequent infectious episodes, whereas neutropenia itself was not (Galloway-Peña et al., 2016).

#### Leukemias, Mainly Acute Myeloid Leukemia, at the Highest Risk

Similarly, as in series studied in our review, the majority of HM patients infected with CR bacteria in case reports had leukemias (19/26, 73%), mostly AML (12/19, 63%; Table 4). Specific alterations in the immunity of leukemia patients and the disease-induced neutropenia for prolonged periods may contribute to the increased risk of CR colonization and infection in this population. Leukemias account for 2.5% of all cancers and affect all aged groups (Bray et al., 2018). Leukemias are usually classified into four categories: acute lymphocytic leukemia (ALL), chronic lymphocytic leukemia (CLL), AML, and chronic myelogenous leukemia (CML). Among lymphocytic leukemias, CLL predominates and mostly concerns the elderly, 70 years being the median age at presentation; while 80% of ALL occur in less than 20 years old people. For unclear reasons, CLL is much rarer in Asian populations than in Western countries where it is the most common leukemia in adults (Rodriguez-Abreu et al., 2007; Sant et al., 2010)^3^.

Myelogenous leukemias are mainly adult diseases with a median age at presentation around 60. The most prevalent is AML, which involves the clonal expansion of myeloid blasts in the bone marrow and peripheral blood (Arber et al., 2016). Globally, AML represents 4 to 6% of HM and 30% of leukemias in adults, with a M/F ratio around 1.4 (World Health Organization, 2014). Incidence of AML increases with age; while the relative survival dramatically reduces with age (50% at 6 months in older than 70 years) (Haematological Malignancy Research Network, 2011). Indeed, patients over 60 years of age more frequently present unfavorable chromosome abnormalities or co-morbid medical conditions that make it more difficult to use intense chemotherapy treatments (Deschler and Lübbert, 2006). There are some worldwide disparities in AML distribution between countries with medium and low human development (World Health Organization, 2014). The AML annual incidence is the highest in Europe [2.5 to 5.5/100 000 age standardized rate (ASR)] and the United States (3.7/100 000 ASR) compared to other areas (0.5 to 3.2/100 000 ASR) (Lubeck et al., 2016). Notably AML is less frequent in Morocco, Yemen, and China and affects younger people in Asia (Chen and Chen, 2014; Errahhali et al., 2016).

**FIGURE 2:**
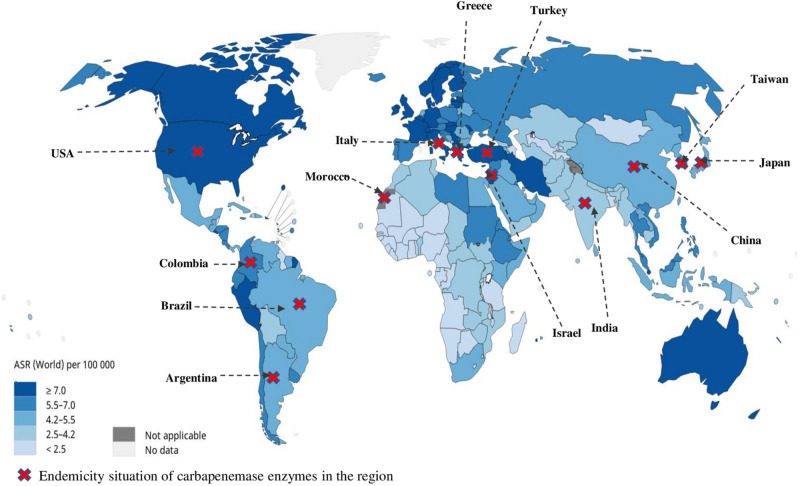
Global repartition of leukemia incidence age-standardized rate provided by the WHO, International Agency for Research on Cancer 2019 (Data source GLOBOCAN 2018 available at http://gco.iarc.fr/today), and the overlay of current endemic distribution of carbapenemases by country.

Unfortunately, no detailed world statistics are available for subclassified leukemias. We overlay the current endemic distribution of carbapenemases by country (Logan and Weinstein, 2017) with the global repartition of leukemia incidence ASR provided by the WHO and the International Agency for Research on Cancer 2019 (Data source GLOBOCAN 2018 available at http://gco.iarc.fr/today) in order to estimate the regions at most risk for CR-bacteria occurrence in hematologic patients (Figure 2). This map brings out the United States, Italy, Greece, Turkey, Israel, and Taiwan as the countries with the highest risk for CR infections in HM patients.

#### Role of Carbapenem Resistance Colonization

Three studies demonstrated that CR carriage was a significant risk factor for CR BSI occurrence in HM patients (Andria et al., 2015; Trecarichi et al., 2016; Jaiswal et al., 2018). The rate of CR-bacteria colonization among HM patients at the admission ranged from 3.8% (85/2226)—in Italy, without association with previous exposure to antibiotics—to 21% (48/225) in India. In this Indian cohort, 20% (46/225) of patients acquired CR colonization during hospitalization (Jaiswal et al., 2018). Of the colonized HM patients, 14% (12/85) developed a subsequent BSI with the same CR germ (Cattaneo et al., 2018). Similarly, in a second study, 18% (17/94) of CR-colonized patients developed CR-bacteria BSIs, all of them during a course of therapy-induced neutropenia (Jaiswal et al., 2018). Likewise, CR-*K. pneumoniae* colonization documented before or after transplant was followed by an infection in 26% of auto-hematopoietic stem cell transplantation (HSCT) and 39% of allo-HSCT CR-colonized patients (Girmenia et al., 2015). AL diagnosis, mostly AML, was the strongest risk factor for overall CR-bacteria colonization and bacteremia occurrence (Ballo et al., 2019).

These data support the idea of implementing prevention strategies based on colonization surveillance and gut decontamination in CR-bacteria carriers with HM. It may be hypothesized that dysbiosis and damage of the mucosal surfaces combined with immunocompromised state associated with HM (mainly AL) and neutropenia increase the risk for CR-Enterobacterial invasive infection in colonized patients. Gut-decolonizing strategies against CR-Enterobacteria include antibiotics, bacteriophage therapy in trials, fecal microbiota transplantation, and alternative treatments such as probiotics or psyllium. Regimens with oral colistin sulphate and/or gentamicin and a regimen with streptomycin and neomycin have been used but with the risk of resistance development and with discordant results in terms of mortality reduction and clinical outcome according to the studies (Tacconelli et al., 2019). Preventive strategies, such as fecal microbiota transplantation, have been reported to be safe and eradication of CR *K. pneumoniae* from the gastrointestinal tract was successful (Bilinski et al., 2017). However, the 2019 ESCMID-EUCIC clinical guidelines do not recommend the routine decolonization of MDR GN bacteria carriers, including immunocompromised carriers, regarding insufficient data for or against any intervention (Tacconelli et al., 2019). Pending on reevaluation of these recommendations, CR-Enterobacteria positive patients should be put under barrier nursing care precautions as per Centers for Disease Control and Prevention guidelines (single room, dedicated nurse and housekeeping staff, contact isolation) (Centers for Disease Control, 2019). Control measures implementation, including weekly rectal screening and contact precautions, reduced significantly the spread of CR *K. pneumoniae* in patients with HM in an Italian teaching hospital (Micozzi et al., 2017).

#### CR in Neutropenic Patients

Neutropenia is a major adverse effect of chemotherapy, HSCT, or disease course in HM patients, mainly AL (Table 3). Neutropenic cases belong to a subgroup of HM patients who are at particularly high risk of infection, including opportunistic and fungal pathogens. In the cohorts of neutropenic HM patients, CR BSI accounted for less than 1% of BSI in Northern Europe (Kjellander et al., 2012), 2% of BSI in New York (Satlin et al., 2016), while 11% and 13% of BSI in China and Cairo, respectively (Table 3; El-Mahallawy et al., 2014; Wang et al., 2015). In the multicentric retrospective American case control study, BSI due to CR bacteria over a 1-year period was of 1.8% in neutropenic HM patients vs. 0.7% in other types of hospitalized patients (*p* = 0.003), and factors independently associated with CR bacteremia in multivariable analyses were the use of a β-lactam/β-lactamase inhibitors or carbapenems within the previous 30 days and treatment with trimethoprim-sulfamethoxazole or glucocorticoids at BSIs onset (Satlin et al., 2016). Likewise, in a meta-analysis of 30 studies, CR in neutropenic patients was associated with previous exposure to carbapenems (OR 4.63, 95% CI [3.08–6.96]), but this result relied on only four papers (13%) that had reported a significant association, none of them being prospective and half (2/4) failing to target HM patients (Righi et al., 2016).

As a result, neutropenic patients undergo multiple concurrent conditions, including antibiotics as prophylaxis or treatment, chemotherapy, and glucocorticoids that may contribute to promote CR-bacteria invasive disease while lacking immune defense mechanisms (Jaiswal et al., 2018).

### Management of CR-Infections in HM Adult Patients

Regarding antimicrobial susceptibility, CR strains in HM patients were globally susceptible to colistin (80–100%), tigecycline (60–80%), or gentamicin (10–60%) (Tables 2−4). The lowest rate of susceptibility to aminoglycosides was recorded in India (Jaiswal et al., 2018) and Italy (Micozzi et al., 2017), where CR-*K. pneumoniae* isolates with high minimal inhibitory concentration to gentamicin, tigecycline, and colistin were reported (Cristina et al., 2017). The worst resistance profile was observed by Micozzi et al. (2017) with only half of CR-*K. pneumoniae* strains susceptible to colistin (12/22), 27% (6/22) to tigecycline, and none (0/22) to gentamicin (Micozzi et al., 2017).

In literature, focusing on CR-*K. pneumoniae*, a “targeted” antibiotic therapy, has been defined as an association of at least two antibiotics, including colistin, tigecycline, and gentamicin, with at least one of them active *in vitro* against the isolate (Tumbarello et al., 2012; Girmenia et al., 2015). The standard therapy required in HM patients with documented CR-bacterial infection is the combination of: tigecycline–gentamicin / colistin–gentamicin / colistin–tigecycline–gentamicin / colistin–tigecycline / all ± in association with meropenem (Table 2). Indeed, the colistin–meropenem combination appeared synergistic *in vitro* against CR bacteria (Paul et al., 2014; Tumbarello et al., 2015; Blennow and Ljungman, 2016; Tofas et al., 2016) but failed to be proven in trials (Paul et al., 2018), and *in vivo* synergisms have not been demonstrated (Del Bono et al., 2016). In a subgroup study, the use of colistin even against colistin-resistant isolates was a cornerstone in the treatment of CR *Acinetobacter* (Dickstein et al., 2018). The clinical efficacy of colistin in combination with rifampicin against colistin-resistant KPC-*K. pneumoniae* and VIM-1-producing *E. cloacae* has also been reported (Tascini et al., 2006, 2013). In ICU nosocomial CR-*K. pneumoniae* BSIs, high-dose regimens of tigecycline (200 mg loading dose followed by 100 mg/12 h) rather than standard dose (100 mg then 50 mg/12 h) were associated with significantly lower mortality without additional adverse effects (Geng et al., 2018).

Combination regimens are efficient in selected patients when antimicrobial susceptibility testing is available, but may have some toxicity, mainly renal. Empiric therapy first-line treatment in patients at-risk of infection with CR bacteria is challenging and could benefit from the arrival of new antibiotics active against CR-Enterobacteria and/or MDR *P. aeruginosa* and *A. baumannii*. However, recommendations and antimicrobial stewardship programs to position these therapeutic options are still lacking. Notably, additional data on clinical efficacy are needed for some of them (Peri et al., 2019). New β-lactamase inhibitors avibactam, relebactam, and vaborbactam can inhibit the activity of KPC-*K. pneumoniae*. Ceftazidime-avibactam was well tolerated and associated with higher clinical cure rates in HM patients with CR-enterobacteremia (Castón et al., 2017). However, its use can be associated with the emergence of resistance in patients with KPC-*K. pneumoniae*, so that it is recommended to associate ceftazidime-avibactam with other antibiotics such as tigecycline, aminoglycoside, colistin, or carbapenem. Moreover, ceftazidime-avibactam and meropenem-vaborbactam may lack activity against MBL (NDM and VIM) (Peri et al., 2019). Cefiderocol (S-649266), a novel siderophore cephalosporin, was stable against relevant carbapenemases, including MBL, KPC, and OXA-48-group carbapenemases. Ceftolozan-tazobactam could be a therapeutic option in non-carbapenemase-producing CR GN strains in HM patients (Saran et al., 2019); Non-β lactam antibiotics, plazomicin and eravacycline, also represent potential options for the treatment of CR GN-bacterial infections. Plazomicin is a new generation aminoglycoside with increased *in vitro* activity against KPC-producing bacteria, but with a limited activity against the NDM group of CR bacteria, and is currently recommended to treat urinary tract infections, but not BSI (Cloutier et al., 2017; Connolly et al., 2018). Eravacycline is a novel fluorocycline structurally similar to tigecycline, not affected by most mechanisms causing tetracycline resistance, and has shown to be active against ESBL, KPC-, and OXA-producing Enterobacteriaceae (Zhanel et al., 2018).

In non-fermenter GN bacteria, colistin in combination with ceftazidime is clinically effective against MDR *P. aeruginosa* (Tascini et al., 2006, 2013). New antimicrobial molecules can inhibit the activity of some non-fermenter GN bacteria. Hakki and Lewis (2018) showed the success of ceftolozane, a novel cephalosporin, used in combination with tazobactam for the treatment of MDR *P. aeruginosa* infections in hematopoietic-cell transplant recipients (Hakki and Lewis, 2018). Unlike in *P. aeruginosa*, eravacycline (the novel fluorocycline) is active against MDR *A. baumannii* and *S. maltophilia* (Zhanel et al., 2018).

It should be noted that active empiric therapy was rarely administrated in HM patients with CR BSIs: 34% in an Israeli study (Andria et al., 2015), 24% in an Italian HCST cohort (Girmenia et al., 2015), and only 14% vs. 56 to 88% in hematological controls with non-CR BSIs (*p* < 0.001) in neutropenic American patients (Satlin et al., 2016; Tables 2−3). For high-risk neutropenic patients, all of the guidelines recommend starting monotherapy with a beta-lactam active against *P. aeruginosa* (piperacillin-tazobactam, imipenem, meropenem, cefepime, ceftazidime) and to add other antimicrobials in patients who are clinically unstable and when there is suspicion of infection caused by resistant GN or GP bacteria (Freifeld et al., 2011; Gea-Banacloche, 2013). But even after susceptibility data, few HM patients received optimal antibiotic therapy. In the cohort of 50 neutropenic HM patients with CR-*K. pneumoniae* BSIs, 90, 86, and 74% of the isolates were susceptible to gentamicin, tigecycline, and colistin, respectively. However, for definitive treatment, 80% received at least one active drug, and only 60% of patients received a combination therapy (Tofas et al., 2016). In the New York series, 80% of CR Enterobacteria were susceptible to colistin, 65% to tigecycline, and 30% to gentamicin, but only 5/15 (33%) received two targeted agents (Satlin et al., 2013a).

**FIGURE 3:**
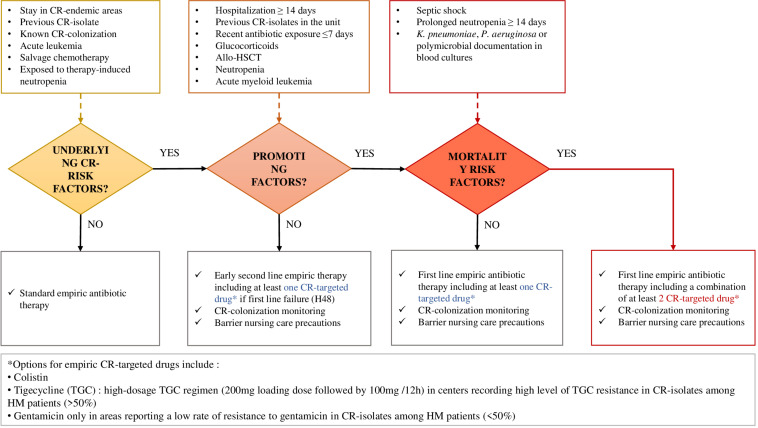
Proposal for CR-bacterial infection risk assessment and empirical management of febrile hematologic patients, based on morbidity and mortality determinants recorded from this literature review.

Therefore, providing adequate treatment for patients with CR bacteria, not only at the time of empirical antibiotic therapy but also after susceptibility test results, remains a real therapeutic challenge. HM patients with CR infection would really benefit from the early help of infectious diseases consultants (Cristina et al., 2017) and should be approached according to the algorithm proposed in Figure 3.

### Factors of Mortality in HM Patients With CR Bacterial Infections

We found the overall mortality rate of HM patients ranged from 7 to 55% according to the series, and mortality was much higher (45 to 100%) among CR-infected HM patients (Tables 2−3). Among CR bacteria, mortality was linked with CR *K. pneumoniae* and CR *A. baumannii*, which were identified as independent risk factors for 30-day mortality in HM patients (Wang et al., 2017). The CR-related mortality rate was particularly high in allo-HSCT patients (64.4% at 3 months) and in neutropenic patients: for example, 51% at 30 days vs. 15% in controls (*p* < 0.001) and 50 vs. 13% in overall BSI-related mortalities (Table 3; Satlin et al., 2013a; Wang et al., 2015; Trecarichi et al., 2016; Kirtane and Lee, 2017). In comparison, in a review of studies published worldwide before 2012, Falagas et al. (2014) estimated that 26 to 44% of deaths worldwide were due to CR (Falagas et al., 2014), and Borer et al. (2009) reported the highest attributable mortality rate for CR-*K. pneumoniae* bacteremia in all comorbidities as 50% with a mortality risk ratio of 3.3 (95% CI, 2.9–28.5) (Borer et al., 2009). Moreover, in a carbapenemase non-endemic European country, CR-colonized HM patients had a significantly reduced 60- and 90-day, as well as 1- and 2-year survival rates when compared to non-colonized patients (Ballo et al., 2019).

It is worth noting that the methods and timing of outcome measurement differ from one study to another and that the age range of the population is often extended across cohorts (Tables 2−3). However, in assessing mortality factors in patients with HM, it is important to take into account the classification and progression of the disease by WHO and the age of the patients, especially in the case of AML where age and neutropenia have a significant impact on survival rates as explained above (Deschler and Lübbert, 2006).

Three studies reported CR-inactive empirical therapy as an independent risk factor of mortality (Falagas et al., 2014; Girmenia et al., 2015; Trecarichi et al., 2016). One study demonstrated inadequate treatment and the absence of active drug combination as a significant risk of death with a mortality rate up to 90% in neutropenic patients with inadequate treatment (Tofas et al., 2016). Likewise, studies investigating intensive care unit patients have demonstrated that the use of combinations with CR *K. pneumoniae*-targeted antibiotics are associated with increased survival of patients with BSIs (Tumbarello et al., 2012). In the Indian prospective series reporting a global infection-related mortality at 26 months as 9.5% (22/225) with 77% of deaths (17/22) due to CR-bacteria BSIs, the mortality in HM patients with CR bacteremia was 100% in a median time of 6 days, with 100% of strains susceptible to colistin and 65% to tigecycline, but the antibiotic therapies and delay of introduction were not specified (Jaiswal et al., 2018). In the Italian multicentric study, the overall 21-day mortality rate in HM patients with bacterial BSIs was significantly higher for patients with BSIs caused by CR Enterobacteriaceae vs. non-CR strains, but no treatment was detailed (Trecarichi et al., 2015). Besides, (Satlin et al., 2013a) reported a 40% mortality rate at 7 days in CR BSI and explained that only 2/18 (11%) of patients had received an adequate empirical therapy. Three deaths occurred before susceptibility data, the median time to introduce an active drug after susceptibility results was 55 h, and only one-third of survivors finally received two active agents representing less than 30% of CR-infected HM patients in this study (Satlin et al., 2013a).

Moreover, in the specific population of neutropenic patients, unresolved neutropenia (i.e., >14 days) was an independent predictor of death (Wang et al., 2015; Tofas et al., 2016). In Jaiswal’s cohort, all CR bacteremia occurred in CR-bacteria carriers during neutropenia, which appears to be a promoting factor of CR-infection occurrence and severity (Jaiswal et al., 2018). Others factor of death were nosocomial acquisition of CR bacteria (Jaiswal et al., 2018) and AML (Micozzi et al., 2017).

Therefore, the delay and the combination of active antimicrobial therapy in HM patients with CR GN bacterial infections, especially if associated with conditions at higher risk of mortality (profound neutropenia, HSCT, AML, and elderly people) exposes them to increased morbidity and mortality. Treatment failures do not appear to be due to a “therapeutic impasse,” but most deaths have occurred in vulnerable HM patients without adequate antibiotic treatments being introduced in time.

## Conclusion and Strategies

As proved with broad-spectrum beta-lactam-resistant Enterobacteria (Woerther et al., 2015), close monitoring CR intestinal carriage can help control their spread and decide on the appropriate initial antimicrobial treatment for vulnerable HM patients with infection. Surveillance of gut colonization in HM populations, at the time of diagnosis and along the course of chemotherapeutic lines and hospitalizations, should be implemented in the most at risk areas we identified (high incidence of carbapenemase bacteria and leukemias), but also in those with a low prevalence of CR to monitor the risk of CR BSI occurrence in HM patients per regions (Ballo et al., 2019).

Importantly, the most at-risk patients should be early identified since the first-line CR-bacteria-targeted treatments do not correspond to standard empiric antibiotic therapies recommended in HM patients, and CR-bacteria identification takes at least 48 h, while time is counted in this group (Sepkowitz, 2005; Averbuch et al., 2013). A critical aspect for the management of CR pathogens in HM patients and the reduction of the related mortality is the definition of early therapeutic strategies based on timely empiric antibiotic combinations, including tigecycline and colistin +/– aminoglycosides, or new drugs such as ceftolozan-tazobactam and ceftazidime-avibactam, according to the antimicrobial susceptibility profiles locally documented. Based on the determinants recorded from this literature review, we propose an algorithm assessing the incremental risk for CR-bacterial infection occurrence and mortality in febrile hematologic patients, guiding the empirical therapeutic strategies (Figure 3).

Finally, the mechanisms (whether it is carbapenemase production or not) and the genetic support of the resistance to carbapenems were poorly documented in the clinical series of HM patients (Satlin et al., 2013a; Girmenia et al., 2015; Tofas et al., 2016). However, the way GN bacteria resist carbapenems may play a role in the antibiotic combination synergies. Clinical trials and studies on therapeutic efficacy should integrate epidemiological data regarding the existing diversity of resistance mechanisms and profiles in CR Enterobacteriaceae (Senchyna et al., 2018).

## Author Contributions

RL and EJ wrote the review manuscript. SB, CU, and J-MR corrected the manuscript. All authors approved and revised the final version of the manuscript.

## Conflict of Interest

The authors declare that the research was conducted in the absence of any commercial or financial relationships that could be construed as a potential conflict of interest.

## Footnotes

1 https://seer.cancer.gov/

2 https://www.hmrn.org/home

3 https://seer.cancer.gov/statfacts/html/clyl.html
